# Developmental stability of general and specific factors of psychopathology from early childhood to adolescence: dynamic mutualism or *p*‐differentiation?

**DOI:** 10.1111/jcpp.12849

**Published:** 2017-12-02

**Authors:** Eoin McElroy, Jay Belsky, Natacha Carragher, Pasco Fearon, Praveetha Patalay

**Affiliations:** ^1^ Institute of Psychology Health and Society University of Liverpool Liverpool UK; ^2^ Department of Human Ecology University of California Davis CA USA; ^3^ Office of Medical Education University of New South Wales Sydney NSW Australia; ^4^ Research Department of Clinical, Educational and Health Psychology University College London London UK

**Keywords:** Comorbidity, continuity, developmental psychopathology, externalizing disorder, internalizing disorder

## Abstract

**Background:**

Recent research indicates that the best‐fitting structural model of psychopathology includes a general factor capturing comorbidity (*p*) and several more specific, orthogonal factors. Little is known about the stability of these factors, although two opposing developmental processes have been proposed: *dynamic mutualism* suggests that symptom‐level interaction and reinforcement may lead to a strengthening of comorbidity (*p)* over time, whereas *p‐differentiation* suggests a general vulnerability to psychopathology that gives way to increasingly distinct patterns of symptoms over time. In order to test both processes, we examine two forms of developmental stability from ages 2 to 14 years: strength (i.e., consistency in the amount of variance explained by general and specific factors) and phenotypic stability (i.e., homotypic and heterotypic continuity).

**Methods:**

Data are from the NICHD Study of Early Child Care and Youth Development. Psychopathology symptoms were assessed nine times between ages 2 and 14 years (*n* = 1,253) using the Child Behavior Checklist completed by mothers. Confirmatory bifactor modeling was used to test structural models of psychopathology at each age. Consistency in strength was examined by calculating the Explained Common Variance (ECV) and phenotypic stability was investigated with cross‐lagged modeling of the general and specific factors.

**Results:**

Bifactor models fit the data well across this developmental period. ECV values were reasonably consistent across development, with the general factor accounting for the majority of shared variance (61%–71%). Evidence of both homotypic and heterotypic continuity emerged, with most heterotypic continuity involving the general factor, as it both predicted and was predicted by specific factors.

**Conclusions:**

A bifactor model effectively captures psychopathological comorbidity from early childhood through adolescence. The longitudinal associations between the general and specific factors provide evidence for *both* the hypothesized processes (*dynamic mutualism* and *p‐differentiation*) occurring through development.

## Introduction

Widespread comorbidity (Kessler et al., 2005) coupled with shared genetic and environmental risk (Waldman, Poore, van Hulle, Rathouz, & Lahey, 2016; Wichstrøm, Belsky, & Steinsbekk, 2017) has led to a surge in models that conceptualize psychopathology as a number of broad transdiagnostic dimensions. Currently the most widely discussed model includes a general psychopathological factor, labeled *p*, that is purported to account for the moderate correlations between psychopathological dimensions (Caspi et al., 2014; Lahey, Krueger, Rathouz, Waldman, & Zald, 2016). In a psychometric sense, *p* is typically represented as a general bifactor that is orthogonally related to a set of specific factors (Caspi et al., 2014; Lahey et al., 2012). As such, *p* explains the variance shared by all psychiatric symptoms/disorders, with additional variance accounted for by distinct specific factors. Specific factors typically take the form of internalizing and externalizing, with additional factors depending on the symptoms/disorders assessed (e.g., thought disorder, attention problems). This model has been replicated in diverse samples of adults (Greene & Eaton, 2017; Lahey et al., 2012) and adolescents/children (Hankin et al., 2017; Laceulle, Vollebergh, & Ormel, 2015; Patalay et al., 2015), using both symptom‐ (Carragher et al., 2016; Patalay et al., 2015) and disorder‐level data (Greene & Eaton, 2017; Laceulle et al., 2015).

Even as *p* is empirically well‐established, it remains a somewhat controversial topic. This model has been criticized for lacking parsimony, and simulation research suggests that fit statistics may be biased toward bifactor models when they are compared with traditional correlated factor models, leading some to suggest that *p* is merely a statistical artifact (Bonifay, Lane, & Reise, 2017). Whether *p* reflects a substantive construct remains open to debate; nevertheless, robust correlations with external variables (e.g., cognitive ability, negative emotionality, demographic factors), and predictive value for future psychopathology (Lahey, Krueger, Rathouz, Waldman, & Zald, 2017; Patalay et al., 2015) suggest that there is at least some utility and informativeness to this general factor.

Despite myriad studies of *p* in recent years, there is little understanding of how it manifests through development and to what degree it changes over time (Murray, Eisner, & Ribeaud, 2016). With regard to its development, two opposing processes have been proposed (Murray et al., 2016). First, ‘*p‐differentiation’* presumes that *p* reflects a general liability to any and all forms of psychopathology, with symptom expression becoming increasingly specific over time (Murray et al., 2016; Patalay et al., 2015). The fact that broad psychopathological dimensions are typically favored earlier in childhood, giving way to more distinct categorical diagnoses as adolescents approach adulthood, in both research and clinical practice, certainly suggests that symptom manifestations become more specific over time (Caspi et al., 2014; Patalay et al., 2015). The alternative perspective, referred to as *dynamic mutualism*, stipulates that, rather than reflecting overall risk, *p* captures local‐level interactions, whereby symptoms directly influence and reinforce each other (Murray et al., 2016; Van Der Maas et al., 2006). When left unchecked, this may result in stronger inter‐item associations over time (Van Der Maas et al., 2006). In consequence, symptoms that are initially distinct may become increasingly associated as psychopathology develops, resulting in increased cross‐domain comorbidity (Caspi et al., 2014; Murray et al., 2016).

Investigation of the developmental stability of *p* may help illuminate its fundamental character, yet few such studies have been reported. Thus, the present report seeks to evaluate and illuminate two types of developmental stability: strength consistency and phenotypic stability. The former refers to the extent that both *p* and specific factors explain a consistent amount of variance over time, whereas the latter refers to the extent to which symptom manifestations remain consistent over development. Both forms of stability may provide insight into the developmental nature of *p*. For example, given that dynamic mutualism is based on the idea that inter‐item correlations become stronger due to reciprocal reinforcement, one would expect to see an increase in the dominant source of covariance (i.e., *p*) over time at the expense of specific factors (Caspi et al., 2014; Murray et al., 2016; Van Der Maas et al., 2006). This would be reflected in greater levels of cross‐domain (i.e., both internalizing and externalizing symptomatology/disorders) comorbidity over development. Alternatively, if *p* represents a more general liability to psychopathology which manifests as increasingly specific expressions over time, one would expect the opposite pattern to occur, with increasingly domain‐specific patterns of psychopathology emerging (Murray et al., 2016; Patalay et al., 2015).

In the only study to have investigated this issue to date, Murray et al. (2016) examined the consistency of the variance explained by *p* in a representative cohort assessed eight times between ages 7 and 15 years (*n *=* *1,572). They found that the variance attributable to both *p* and specific factors remained consistent over time, with *p* accounting for the vast majority of shared variance. Notably, the conclusions drawn from these results were that neither dynamic mutualism nor *p*‐differentiation alone were sufficient to explain the development of comorbidity over this period, as their hypothesized patterns of increasing or decreasing symptom covariance attributable to *p* were not observed. Indeed, it remains possible that both dynamic processes are at play and, when examined solely using a variance explained approach, this may result in the two opposing processes effectively cancelling each other out. This possibility is explicitly examined in the current study, as we investigate the phenotypic stability of *p* and specific psychopathological factors.

Phenotypic stability is perhaps best thought of in terms of homotypic continuity (i.e., one construct predicting itself at a later time point) and heterotypic continuity (i.e., one construct predicting *another* construct at a later time point) (Angold, Costello, & Erkanli, 1999). Evidence of heterotypic continuity could be consistent with dynamic mutualism or *p*‐differentiation, depending on the direction of the effects. A positive association between *p* at baseline and a specific factor at a later time point would be in line with *p*‐differentiation, with a general manifestation of comorbid psychopathology placing an individual at risk of more specific manifestations later on. Alternatively, positive associations between specific factors at baseline and subsequent *p* would suggest that individuals suffering from a particular type of psychopathology may be more likely to develop cross‐domain comorbidity over time as intersymptom associations become stronger; this would be consistent with dynamic mutualism.

To our knowledge, only two studies have examined the phenotypic stability of *p*. Greene and Eaton (2017) examined the homotypic and heterotypic continuity of *p,* as well as the specific internalizing and externalizing dimensions in the National Epidemiologic Survey on Alcohol and Related Conditions (age range 18–90 +  years). Strong homotypic continuity was observed between waves 1 and 2, with no significant heterotypic continuity, a result consistent with Snyder, Young, and Hankin's (2017) study of young adolescents (i.e., between 13.5 and 15 years of age). Such results suggest, once again, that neither dynamic mutualism nor *p*‐differentiation adequately characterizes the development of *p*. It must be noted, however, that both studies just cited were limited to only two assessment waves, with little time (approximately 18–24 months) between assessments. A more extensive longitudinal investigation, especially one covering a time when developmental changes are more salient and symptom stability is in greater flux, might provide a clearer picture of the phenotypic stability of *p* and specific factors across development.

The present study aimed to extend current understanding of the development of *p* in three ways. First, we examine the structure of psychopathology in a sample assessed nine times from early childhood (age 2 years) through adolescence (age 14 years). In light of the fact that most work on the *p*‐factor model using preadult samples has focused on adolescents (Hankin et al., 2017; Laceulle et al., 2015; Martel et al., 2017; Patalay et al., 2015; Snyder, Young, & Hankin, 2016), although one study includes children as young as 3 years of age (Olino, Dougherty, Bufferd, Carlson, & Klein, 2014), we are positioned to determine not only whether the model holds for very young children but also how it develops over childhood and into early adolescence. Moreover, the research reported herein evaluates the consistency over time of the strength of the *p* factor and specific factors in terms of the variance they account for across this developmental period. The only previous study to examine this aspect of *p* began at age 7 (Murray et al., 2016), potentially missing out on a key period over which the processes that underlie comorbidity may develop. Finally, the current work chronicles phenotypic stability, that is, the homotypic and heterotypic continuity of *p* and specific factors over time.

## Methods

### Sample/Participants

Data came from the National Institute of Child Health and Human Development Study of Early Child Care and Youth Development, a prospective cohort study of children born in 1991 at 10 locations across the United States (NICHD Early Child Care Research Network, 2005). The initial sample comprised of 1,364 parent–child pairs. For the present study, data were available for 1,253 children (51% female). Although the sample was diverse, it was not designed to be nationally representative, in that participating families had higher average income and education and were less likely to be of an ethnic minority (Watamura, Phillips, Morrissey, McCartney, & Bub, 2011). Ethical approval for the NICHD study was granted by all data‐collecting universities prior to data collection and at each assessment informed consent was secured from parents and/or teacher. More detailed descriptions of the NICHD Study, including recruitment and assessment procedures, are available elsewhere (NICHD Early Child Care Research Network, 2005).

### Measures

Psychopathology was measured using the Child Behavior Checklist (CBCL) (Achenbach & Rescorla, 2001), a standardized parent report completed by mothers at ages 2, 3, 5, 6, 8, 9, 10, 11, and 14 years. The CBCL assesses 113 psychopathology symptoms, each of which is rated on a 3‐point scale (0 = not true; 1 = somewhat/sometimes true; 2 = very true/often). For 2–3 year olds, the CBCL/1.5–5 was used (Achenbach, Edelbrock, & Howell, 1987). The CBCL/6–18 (Achenbach & Rescorla, 2001), was used to assess problems from age 5 years on. The CBCL/6–18 is a revised version of the CBCL/4–18 (Achenbach, 1991), in which certain items were replaced (for a description of these differences see Achenbach & Rescorla, 2001). For consistency, the present study only included items that were present in both the CBCL/4–18 and CBCL/6–18 measures from age 5 through to age 14; thus, we omitted the additional CBCL/6–18 items that were added/replaced from age 10. Items from the two broad dimensions of internalizing and externalizing, along with items from the distinct attention problems syndrome were included in the present analysis. Due to low endorsement of responses indicating severe presence of symptoms, items were rescored to indicate presence of symptoms (i.e., raw responses of 1 or 2 coded as 1) in line with common practice when conducting item‐level analyses of the CBCL (Achenbach & Rescorla, 2001; Deutz, Geeraerts, van Baar, Deković, & Prinzie, 2016). To aid estimation, items with minimal variation (i.e., counts of five individuals or less in either of the binary categories) were removed.

### Missing data

In order to ensure that attrition did not bias the results, imputation was conducted using the *R* package AMELIA‐II, which uses expectation‐maximization (Honaker, King, & Blackwell, 2011). A single imputed dataset based on all participants who provided at least some CBCL data was produced (*n* *=* 1,253).

### Statistical analysis

First, confirmatory factor models (CFAs) and confirmatory bifactor models (CBMs) were estimated at each time point. The CFAs were comprised of three correlated factors: internalizing, externalizing and attention problems. The CBMs included a general factor that was uncorrelated with the three specific factors. The correlations between the specific factors were fixed to zero in the bifactor models. Bifactor models were chosen over second‐order factor models as they were deemed a more appropriate means of testing dynamic mutualism versus *p*‐differentiation. With second‐order modeling, the general factor represents the variance shared by the specific factors. Bifactor models differ in that the general and specific factors exist at the same theoretical level and compete to explain item variance; thus, the variance explained by both general and specific factors can be directly compared.

There is some debate as to whether attention/hyperactivity problems form a distinct psychopathological dimension, or whether they should be considered part of the externalizing dimension (Krueger & South, 2009). The developmental literature has largely treated attention/hyperactivity as a unique dimension that correlates with both internalizing and externalizing (Achenbach & Rescorla, 2001; Deutz et al., 2016; Geeraerts et al., 2015). Given that the CBCL makes this distinction, a distinct attention/hyperactivity factor was included in the present analysis.

Confirmatory factor models and confirmatory bifactor models were estimated using the robust weighted least squares estimator (WLSMV). Models were identified by fixing the variance of each factor to 1, and freely estimating the first factor loading. Model fit was assessed using the Comparative Fit Index (CFI) and Tucker Lewis Index (TLI) for which, respectively, values of >.90 reflect acceptable fit and >.95 represent very good model fit (Hu & Bentler, 1999; Little, 2013), and the root mean‐square error of approximation (RMSEA) (Steiger, 1990), for which values below .06 are considered acceptable (Hu & Bentler, 1999). Construct reliability (*H*) was assessed based on the guidelines of Hancock and Mueller (2001). *H* evaluates how well a latent variable is represented by its given items, and as such how suitable and replicable an *SEM* model is likely to be (Rodriguez, Reise, & Haviland, 2016). *H* is calculated as the ratio of variance explained by a latent variable relative to the variance left unexplained. Ranging from 0 to 1, higher values indicate better defined latent variables, with values greater than .8 deemed well‐defined and likely stable across studies (Hancock & Mueller, 2001).

### Strength consistency

To compare the relative strength of both the general and specific factors across time, the explained common variance (ECV) for each factor was calculated at each time point. The ECV was developed to test the unidimensionality of a psychometric scale and is calculated by dividing the variance explained by the factor of interest by the variance explained by the general and specific factors combined. ECV values can also be calculated in a similar manner for the specific factors. ECV values range from 0 to 1, with values closer to 1 suggesting a greater share of variance explained. There are no established cutoffs in ECV values; it has been suggested that ECV values ranging from .6 (Reise, Scheines, Widaman, & Haviland, 2013) to .85 (Reise & Revicki, 2014) indicate that a factor is the main source of shared variance.

### Phenotypic stability

Homotypic and heterotypic continuity of the general and specific factors were examined using cross‐lagged panel analysis. Because a single structural equation model was not possible due to the number of parameters involved, a ‘two‐step’ approach was adopted. First, factor models were specified and estimated at each age and factor scores saved. Second, these factor scores were used as observed variables in the cross‐lagged regression model (Figure 2). This model was estimated using robust maximum likelihood (MLR) in Mplus 7.4 (Muthén & Muthén, 2012).

## Results

First presented are results of the overall model fit, followed by those pertaining to strength consistency and, thereafter, phenotypic stability.

### Overall model fit, factor loadings, and reliability

The fit statistics for the correlated factor and bifactor models are presented in Table 1. The CFAs mostly indicated adequate fit, with the one exception being the correlated model at the 3‐year time point (TLI < .90). The bifactor models all demonstrated adequate‐to‐good fit (Little, 2013).

**Table 1:** Fit Statistics of CFA and bifactor models across time

Age (years)	Model	χ^2^	*df*	CFI	TLI	RMSEA
2	Correlated	3,542.14*	1,707	.90	.90	.03
	Bifactor	2,874.90*	1,650	.93	.93	.02
3	Correlated	3,701.98*	1,707	.90	.89	.03
	Bifactor	3,138.69*	1,650	.92	.92	.03
5	Correlated	3,634.13*	2,141	.91	.90	.02
	Bifactor	3,146.60*	2,077	.93	.93	.02
6	Correlated	3,421.18*	2,076	.91	.91	.02
	Bifactor	3,095.54*	2,013	.93	.92	.02
8	Correlated	3,876.13*	2,076	.91	.90	.03
	Bifactor	3,280.58*	2,013	.94	.93	.02
9	Correlated	3,409.85*	2,012	.92	.91	.02
	Bifactor	2,977.06*	1,950	.94	.93	.02
10	Correlated	3,651.75*	2,076	.91	.90	.03
	Bifactor	3,227.41*	2,013	.93	.92	.02
11	Correlated	3,760.60*	2,141	.91	.90	.03
	Bifactor	3,376.48*	2,142	.93	.93	.02
14	Correlated	3,754.89*	2,342	.92	.92	.02
	Bifactor	3,321.80*	2,275	.94	.94	.02

*p < .01.

Means and standard deviations for the scales, standardized factor loadings, and construct reliability values (*H*) at each age are presented in the online supplementary materials (Tables S1–S9). *H* values were highest for *p*, ranging from .95 to .97. *H* values were also consistently above .8 for internalizing. This suggests both *p* and internalizing factors were well represented by their items (Hancock & Mueller, 2001). *H* values ranged from .70 to .82 for externalizing and .59 to .68 for attention, suggesting these factors were less well‐defined (Hancock & Mueller, 2001).

### Strength consistency

**Figure 1:**
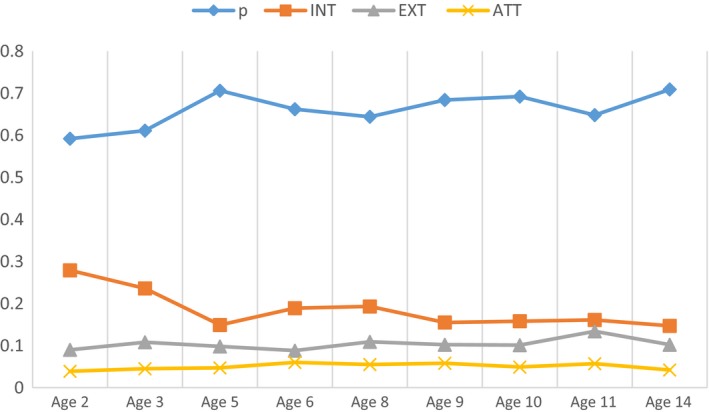
ECV values across time. *p,* general psychopathology; INT, internalizing; EXT, externalizing; ATT, attention problems [Colour figure can be viewed at http://wileyonlinelibrary.com]

Figure 1 displays ECV values across time. Despite some minor fluctuation in magnitude, *p* explained the vast majority of variance at all time points (.60–.71) (Reise et al., 2013; Reise & Revicki, 2014).

Of the three specific factors, internalizing explained the most additional variance at each time point (.14–.25). The attention factor explained little overall variance (.04–.06). Overall, ECV values did not consistently exceed .70, suggesting that although *p* explained the majority of variance, the specific factors explained a nontrivial amount of variance across time (Rodriguez et al., 2016).

### Phenotypic stability

**Figure 2:**
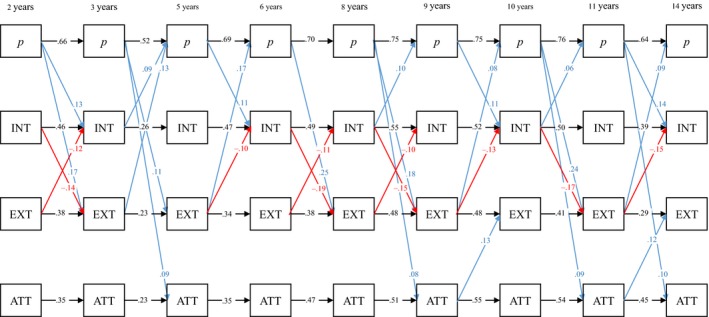
Standardized path coefficients (calculated using factor scores) across time. All paths significant at *p* < .01. *p,* general psychopathology; INT, internalizing; EXT, externalizing; ATT, attention problems [Colour figure can be viewed at http://wileyonlinelibrary.com]

Standardized regression coefficients based on latent factor scores are presented in Figure 2. All autoregressive (i.e., homotypic) effects were statistically significant. These autoregressive effects were consistently largest for *p* (β* *= .52–.76), with effects for specific factors ranging from small to moderate (β* *= .23–.55). Externalizing and attention evidenced the least phenotypic stability. This may have been partly due to their lower levels of construct reliability (*H*) (Hancock & Mueller, 2001).

Notably, a large number of cross‐lagged effects were statistically significant, although these were considerably weaker than the homotypic autoregressive effects (β* *= .06–.25). Nevertheless, consistent patterns emerged. Internalizing and externalizing frequently had negative associations across time (β = −.10 to −.19). *p* positively predicted the three specific factors at varying points across the timeframe (β = .08–.25), while itself being predicted by both internalizing (β = .06–.10) and externalizing (β = .08–.17). To determine whether certain heterotypic paths were significantly stronger than others, equality constraints were placed on pathways. A Wald test indicated that there was a significant difference between paths leading from internalizing and externalizing to *p*, respectively (Wald test Σ^2^ (8) = 98.99, *p* < .01). Similarly, paths from *p* to internalizing and externalizing were significantly different (Wald test Σ^2^ (8) = 100.93, *p* < .01). Given the larger standardized effect sizes between *p* and externalizing, it appears these two factors were more strongly associated over time than *p* and internalizing.

## Discussion

The present study sought to illuminate the structure and developmental stability of psychopathology in a cohort of children assessed repeatedly from very early childhood to midadolescence. This research was based on the *p‐*factor approach to the modeling of psychopathology, with a general bifactor accounting for the covariance between all psychopathological symptoms (Caspi et al., 2014; Lahey et al., 2012). Although *p* has repeatedly been discerned in cross‐sectional data, few studies have examined its development. Investigating the longitudinal development of general and specific dimensions of psychopathology may lead to a better understanding of the development of psychiatric disorders across the life course. Furthermore, a better understanding of the developmental processes that underlie *p* may help establish an agreed‐upon interpretation of this factor, something that has so far proven elusive.

The present study examined two forms of developmental stability, strength consistency, and phenotypic stability, in an attempt to determine whether the development of *p* was characterized by either dynamic mutualism or *p*‐differentiation. With regard to consistency in strength, both *p* and the specific factors explained very consistent proportions of variance across time, with *p* explaining the majority of shared variance at all time points. These findings are in line with those of Murray et al. (2016). The fact that the amount of variance accounted for by *p* and the specific factors did not change over time seems inconsistent with both *p*‐differentiation and dynamic mutualism, as they hypothesize respectively, increasing and decreasing relative contributions of *p* to symptom variance across time. It must be noted, however, that *p* was derived from cross‐sectional data at each time point in the present study and that of Murray et al. (2016), meaning each *p* factor represented the variance attributable to comorbidity, in whatever form that took, at a particular age. In simple terms, *p* was used to capture all comorbidity at a given age, although the underlying meaning of *p* may vary developmentally.

Heterotypic continuity was notably evident, even if it was less common and weaker than homotypic continuity. The presence of heterotypic continuity suggests significant individual‐level change in the phenotypic expression of psychopathology. Overall, *p* appeared to lie at the heart of heterotypic continuity, with almost all positive heterotypic effects feeding into or emerging from *p*. For example, *p* predicted all three specific factors at subsequent time points, and was itself predicted at future time points at least once by earlier measurements of both externalizing and internalizing. This suggests that specific manifestations of psychopathology led to increased risk of comorbid psychopathology (i.e., *p*) over time, and vice versa. This would seem to contradict the previous claim that *neither* dynamic mutualism nor *p*‐differentiation characterizes the development of psychopathology. Indeed, the data suggest that both developmental processes may be operating concurrently, possibly cancelling each other out when using the relative amount of variance explained as a measure of stability (e.g., Murray et al., 2016). Put simply, there may be certain individuals for whom symptoms become more specific over time (i.e., *p →* internalizing, externalizing, or attention), whereas others experience more comorbidity (internalizing, externalizing, or attention *→ p*), and still others who experience both processes.

Having raised the aforementioned possibilities, a closer examination of the individual effects observed herein would seem in line with previous studies using person‐centered methodologies, which further support the idea that both processes, *p*‐differentiation and dynamic mutualism, may occur simultaneously. For example, person‐centered studies have identified unique classes that describe patterns of co‐occurring symptomatology both cross‐sectionally (Vaidyanathan, Patrick, & Iacono, 2011) and longitudinally through childhood (Patalay, Moulton, Goodman, & Ploubidis, 2017). Recent research investigating transitions between these cross‐sectional classes over time indicate that transitions between ‘pure’ psychopathological classes (e.g., predominantly internalizing or externalizing) and comorbid classes are relatively common, with transitions more often observed to and from comorbid and predominantly externalizing classes (Basten et al., 2016; Kim & Eaton, 2017; McElroy, Shevlin, & Murphy, 2017; Willner, Gatzke‐Kopp, & Bray, 2016). The findings of the present variable‐centered study mirror these results; externalizing and *p* demonstrated stronger reciprocal relationships over time than internalizing and *p*. Indeed, it appears that those who score high on *p* initially could potentially develop more specific psychopathology in any of the three specific domains. Those with more externalizing problems at baseline, however, appear more likely to develop comorbid internalizing symptoms over time, whereas the converse is less likely. This is very much in line with cascade models, which have highlighted the role of externalizing in the development of subsequent co‐occurring internalizing problems (Capaldi, 1992; Masten & Cicchetti, 2010).

To date, only two other investigations have examined the phenotypic stability of general and specific factors of psychopathology, with both chronicling strong homotypic continuity but not heterotypic continuity (Greene & Eaton, 2017; Snyder et al., 2016). It must be noted, however, that Greene and Eaton (2017) utilized an adult sample, for which a greater degree of phenotypic stability would be expected compared with samples of children/adolescents. Furthermore, both studies assessed psychopathology at only two time points, with a limited amount of time between assessments. The present investigation discerned heterotypic continuity over multiple time points, across a period of significant development. As such, it indicates that processes such as *p*‐differentiation and/or dynamic mutualism are more pronounced earlier in development.

### Strengths and limitations

A major strength of the present study was the large developmental range covered, from very early childhood to midadolescence. Early and middle childhood are dynamic periods of the life span, involving significant biological, cognitive, and social change, and it is well‐established that psychopathology in childhood is a precursor to mental ill‐health in adulthood (Kessler et al., 2007). With regard to limitations, first it must be noted that the present study relied solely on maternal reports across all assessment waves, however, proxy reports are considered necessary in early childhood. It is worth noting that when children become old enough to self‐report, cross‐informant agreement has generally been low (Waters, Stewart‐Brown, & Fitzpatrick, 2003). Second, while the sample used in the present study was large and diverse, it was not nationally representative. Third, due to the computational complexity of the tested model, the preferred method of employing a single longitudinal structural equation model was not possible. The implemented ‘two‐step’ approach has some weaknesses (e.g., smaller standard errors, biased estimates) (DiStefano, Zhu, & Mindrila, 2009). Finally, as previously mentioned, the longitudinal invariance of *p* was not tested in the present study, meaning *p* in this case reflected only a broad statistical summary of comorbidity across internalizing, externalizing, and attention problems.

## Conclusion

The current study highlights the utility of a general factor as a method for capturing psychopathological comorbidity and investigating the role that comorbidity plays in the development of mental illness. Contrary to prior research, the findings demonstrated that *p*‐differentiation and dynamic mutualism are both plausible mechanisms by which psychopathology develops over time. It is possible that there are specific pathways experienced by different individuals across development. Further studies implementing both variable‐centered and person‐centered techniques may be required to better illuminate these developmental pathways, differentiate those who experience them, and examine the outcomes associated with each.

**Key points**

- Research suggests that a bifactor model comprised of a general bifactor and specific factors effectively captures psychiatric comorbidity across development, yet the developmental stability of these factors remains under‐researched.
- Confirmatory bifactor modeling demonstrated that this model fit well in a cohort of children assessed between ages 2 and 14 years, with the general factor accounting for the vast majority of symptom covariation.
- Cross‐lagged modeling indicated that this general factor temporally predicted and was predicted by specific factors.
- Psychopathological comorbidity may become increasingly/decreasingly distinct over time, or demonstrate intermittent periods of both.

## Supporting information


**Table S1.** Means (*SD*), construct reliability (*H*), and standardized factor scores at age 2.
**Table S2.** Means (*SD*), construct reliability (*H*), and standardized factor scores at age 3.
**Table S3.** Means (*SD*), construct reliability (*H*), and standardized factor scores at age 5.
**Table S4.** Means (*SD*), construct reliability (*H*), and standardized factor scores at age 6.
**Table S5.** Means (*SD*), construct reliability (*H*), and standardized factor scores at age 8.
**Table S6.** Means (*SD*), construct reliability (*H*), and standardized factor scores at age 9.
**Table S7.** Means (*SD*), construct reliability (*H*), and standardized factor scores at age 10.
**Table S8.** Means (*SD*), construct reliability (*H*), and standardized factor scores at age 11.
**Table S9.** Means (*SD*), construct reliability (*H*), and standardized factor scores at age 14.

## References

[jcpp12849-bib-0002] Achenbach, T.M. (1991). Child behaviour checklist and related material (1st edn). Burlington, VT: University of Vermont.

[jcpp12849-bib-0003] Achenbach, T.M. , Edelbrock, C. , & Howell, C.T. (1987). Empirically based assessment of the behavioral/emotional problems of 2‐and 3‐year‐old children. Journal of Abnormal Child Psychology, 15, 629–650. 3437096 10.1007/BF00917246

[jcpp12849-bib-0004] Achenbach, T.M. , & Rescorla, L.A. (2001). Manual for the ASEBA School‐Age Forms & Profiles. Burlington: VT: University of Vermont, Research Center for Children, Youth, & Families.

[jcpp12849-bib-0005] Angold, A. , Costello, E.J. , & Erkanli, A. (1999). Comorbidity. Journal of Child Psychology and Psychiatry, 40, 57–87. 10102726

[jcpp12849-bib-0006] Basten, M. , Tiemeier, H. , Althoff, R.R. , van de Schoot, R. , Jaddoe, V.W. , Hofman, A. , … & van der Ende, J. (2016). The stability of problem behavior across the preschool years: An empirical approach in the general population. Journal of Abnormal Child Psychology, 44, 393–404. 25832625 10.1007/s10802-015-9993-y PMC4729812

[jcpp12849-bib-0007] Bonifay, W. , Lane, S.P. , & Reise, S.P. (2017). Three concerns with applying a bifactor model as a structure of psychopathology. Clinical Psychological Science, 5, 184–186.

[jcpp12849-bib-0008] Capaldi, D.M. (1992). Co‐occurrence of conduct problems and depressive symptoms in early adolescent boys: II. A 2‐year follow‐up at Grade 8. Development and Psychopathology, 4, 125–144. 10.1017/s0954579499001959 10208356

[jcpp12849-bib-0009] Carragher, N. , Teesson, M. , Sunderland, M. , Newton, N. , Krueger, R. , Conrod, P. , … & Slade, T. (2016). The structure of adolescent psychopathology: A symptom‐level analysis. Psychological Medicine, 46, 981–994. 26620582 10.1017/S0033291715002470

[jcpp12849-bib-0010] Caspi, A. , Houts, R.M. , Belsky, D.W. , Goldman‐Mellor, S.J. , Harrington, H. , Israel, S. , … & Poulton, R. (2014). The p factor: One general psychopathology factor in the structure of psychiatric disorders? Clinical Psychological Science, 2, 119–137. 25360393 10.1177/2167702613497473 PMC4209412

[jcpp12849-bib-0011] Deutz, M.H. , Geeraerts, S.B. , van Baar, A.L. , Deković, M. , & Prinzie, P. (2016). The Dysregulation Profile in middle childhood and adolescence across reporters: Factor structure, measurement invariance, and links with self‐harm and suicidal ideation. European Child and Adolescent Psychiatry, 25, 431–442. 26226917 10.1007/s00787-015-0745-x PMC4820491

[jcpp12849-bib-0012] DiStefano, C. , Zhu, M. , & Mindrila, D. (2009). Understanding and using factor scores: Considerations for the applied researcher. Practical Assessment, Research and Evaluation, 14, 1–11.

[jcpp12849-bib-0013] Geeraerts, S.B. , Deutz, M.H.F. , Deković, M. , Bunte, T. , Schoemaker, K. , Espy, K.A. , … & Matthys, W. (2015). The Child Behavior Checklist Dysregulation Profile in preschool children: A broad dysregulation syndrome. Journal of the American Academy of Child and Adolescent Psychiatry, 54, 595–602. e592. 26088665 10.1016/j.jaac.2015.04.012

[jcpp12849-bib-0014] Greene, A.L. , & Eaton, N.R. (2017). The temporal stability of the bifactor model of comorbidity: An examination of moderated continuity pathways. Comprehensive Psychiatry, 72, 74–82. 27764677 10.1016/j.comppsych.2016.09.010

[jcpp12849-bib-0015] Hancock, G. , & Mueller, R. (2001). Rethinking construct reliability within latent variable systems In Cudeck R., du Toit S. & Sorbom D. (Eds.), Structural equation modeling: Present and future—A Festschrift in honor of Karl Joreskog (pp. 195–216). Lincolnwood, IL: Scientific Software International.

[jcpp12849-bib-0016] Hankin, B.L. , Davis, E.P. , Snyder, H. , Young, J.F. , Glynn, L.M. , & Sandman, C.A. (2017). Temperament factors and dimensional, latent bifactor models of child psychopathology: Transdiagnostic and specific associations in two youth samples. Psychiatry Research, 252, 139–146. 28264785 10.1016/j.psychres.2017.02.061 PMC5439427

[jcpp12849-bib-0017] Honaker, J. , King, G. , & Blackwell, M. (2011). Amelia II: A program for missing data. Journal of Statistical Software, 45, 1–47.

[jcpp12849-bib-0018] Hu, L.T. , & Bentler, P.M. (1999). Cutoff criteria for fit indexes in covariance structure analysis: Conventional criteria versus new alternatives. Structural Equation Modeling: A Multidisciplinary Journal, 6, 1–55.

[jcpp12849-bib-0019] Kessler, R.C. , Amminger, G.P. , Aguilar‐Gaxiola, S. , Alonso, J. , Lee, S. , & Ustun, T.B. (2007). Age of onset of mental disorders: A review of recent literature. Current Opinion in Psychiatry, 20, 359. 17551351 10.1097/YCO.0b013e32816ebc8c PMC1925038

[jcpp12849-bib-0020] Kessler, R.C. , Berglund, P. , Demler, O. , Jin, R. , Merikangas, K.R. , & Walters, E.E. (2005). Lifetime prevalence and age‐of‐onset distributions of DSM‐IV disorders in the National Comorbidity Survey Replication. Archives of General Psychiatry, 62, 593–602. 15939837 10.1001/archpsyc.62.6.593

[jcpp12849-bib-0021] Kim, H. , & Eaton, N.R. (2017). A hierarchical integration of person‐centered comorbidity models: Structure, stability, and transition over time. Clinical Psychological Science, 5, 595–612.

[jcpp12849-bib-0024] Krueger, R.F. , & South, S. (2009). Externalizing disorders: Cluster 5 of the proposed meta‐structure for DSM‐V and ICD‐11. Psychological Medicine, 39, 2061–2070. 19796431 10.1017/S0033291709990328

[jcpp12849-bib-0025] Laceulle, O.M. , Vollebergh, W.A. , & Ormel, J. (2015). The structure of psychopathology in adolescence replication of a general psychopathology factor in the TRAILS Study. Clinical Psychological Science, 3, 850–860.

[jcpp12849-bib-0026] Lahey, B.B. , Applegate, B. , Hakes, J.K. , Zald, D.H. , Hariri, A.R. , & Rathouz, P.J. (2012). Is there a general factor of prevalent psychopathology during adulthood? Journal of Abnormal Psychology, 121, 971. 22845652 10.1037/a0028355 PMC4134439

[jcpp12849-bib-0027] Lahey, B.B. , Krueger, R.F. , Rathouz, P.J. , Waldman, I.D. , & Zald, D.H. (2016). A hierarchical causal taxonomy of psychopathology across the life span. Psychological Bulletin, 143, 142–186. 28004947 10.1037/bul0000069 PMC5269437

[jcpp12849-bib-0028] Lahey, B.B. , Krueger, R.F. , Rathouz, P.J. , Waldman, I.D. , & Zald, D.H. (2017). Validity and utility of the general factor of psychopathology. World Psychiatry, 16, 142–144. 28498590 10.1002/wps.20410 PMC5428167

[jcpp12849-bib-0029] Little, T.D. (2013). Longitudinal structural equation modeling. New York: Guilford Press.

[jcpp12849-bib-0030] Martel, M.M. , Pan, P.M. , Hoffmann, M.S. , Gadelha, A. , do Rosário, M.C. , Mari, J.J. , … & Bressan, R.A. (2017). A general psychopathology factor (P factor) in children: Structural model analysis and external validation through familial risk and child global executive function. Journal of Abnormal Psychology, 126, 137. 27748619 10.1037/abn0000205

[jcpp12849-bib-0031] Masten, A.S. , & Cicchetti, D. (2010). Developmental cascades. Development and Psychopathology, 22, 491. 20576173 10.1017/S0954579410000222

[jcpp12849-bib-0032] McElroy, E. , Shevlin, M. , & Murphy, J. (2017). Internalizing and externalizing disorders in childhood and adolescence: A latent transition analysis using ALSPAC data. Comprehensive Psychiatry, 75, 75–84. 28334631 10.1016/j.comppsych.2017.03.003

[jcpp12849-bib-0033] Murray, A.L. , Eisner, M. , & Ribeaud, D. (2016). The development of the general factor of psychopathology ‘p factor'through childhood and adolescence. Journal of Abnormal Child Psychology, 44, 1573–1586. 26846993 10.1007/s10802-016-0132-1

[jcpp12849-bib-0034] Muthén, L.K. , & Muthén, B.O. (2012). Mplus. Statistical analysis with latent variables. Version, 7.

[jcpp12849-bib-0035] NICHD Early Child Care Research Network (2005). Child care and child development: Results from the NICHD study of early child care and youth development. New York: Guilford Press.

[jcpp12849-bib-0036] Olino, T.M. , Dougherty, L.R. , Bufferd, S.J. , Carlson, G.A. , & Klein, D.N. (2014). Testing models of psychopathology in preschool‐aged children using a structured interview‐based assessment. Journal of Abnormal Child Psychology, 42, 1201–1211. 24652485 10.1007/s10802-014-9865-x PMC4229848

[jcpp12849-bib-0037] Patalay, P. , Fonagy, P. , Deighton, J. , Belsky, J. , Vostanis, P. , & Wolpert, M. (2015). A general psychopathology factor in early adolescence. The British Journal of Psychiatry, 207, 15–22. 25906794 10.1192/bjp.bp.114.149591

[jcpp12849-bib-0038] Patalay, P. , Moulton, V. , Goodman, A. , & Ploubidis, G.B. (2017). Cross‐domain symptom development typologies and their antecedents: Results from the UK millennium cohort study. Journal of the American Academy of Child and Adolescent Psychiatry, 56, 765–776. e762. 28838581 10.1016/j.jaac.2017.06.009

[jcpp12849-bib-0039] Reise, S.P. , & Revicki, D.A. (2014). Handbook of item response theory modeling: Applications to typical performance assessment. Abingdon, UK: Routledge.

[jcpp12849-bib-0040] Reise, S.P. , Scheines, R. , Widaman, K.F. , & Haviland, M.G. (2013). Multidimensionality and structural coefficient bias in structural equation modeling: A bifactor perspective. Educational and Psychological Measurement, 73, 5–26.

[jcpp12849-bib-0041] Rodriguez, A. , Reise, S.P. , & Haviland, M.G. (2016). Applying bifactor statistical indices in the evaluation of psychological measures. Journal of Personality Assessment, 98, 223–237. 26514921 10.1080/00223891.2015.1089249

[jcpp12849-bib-0042] Snyder, H.R. , Young, J.F. , & Hankin, B.L. (2016). Strong homotypic continuity in common psychopathology‐, internalizing‐, and externalizing‐specific factors over time in adolescents. Clinical Psychological Science, 5, 98–110. 28239532 10.1177/2167702616651076 PMC5320894

[jcpp12849-bib-0043] Steiger, J.H. (1990). Structural model evaluation and modification: An interval estimation approach. Multivariate Behavioral Research, 25, 173–180. 26794479 10.1207/s15327906mbr2502_4

[jcpp12849-bib-0606] Snyder, H.R. , Young, J.F. , & Hankin, B.L. (2017). Strong homotypic continuity in common psychopathology‐, internalizing‐, and externalizing‐specific factors over time in adolescents. Clinical Psychological Science, 5, 98–110. 28239532 10.1177/2167702616651076 PMC5320894

[jcpp12849-bib-0044] Vaidyanathan, U. , Patrick, C.J. , & Iacono, W.G. (2011). Patterns of comorbidity among mental disorders: A person‐centered approach. Comprehensive Psychiatry, 52, 527–535. 21111407 10.1016/j.comppsych.2010.10.006 PMC3110544

[jcpp12849-bib-0045] Van Der Maas, H.L. , Dolan, C.V. , Grasman, R.P. , Wicherts, J.M. , Huizenga, H.M. , & Raijmakers, M.E. (2006). A dynamical model of general intelligence: The positive manifold of intelligence by mutualism. Psychological Review, 113, 842. 17014305 10.1037/0033-295X.113.4.842

[jcpp12849-bib-0046] Waldman, I.D. , Poore, H.E. , van Hulle, C. , Rathouz, P.J. , & Lahey, B.B. (2016). External validity of a hierarchical dimensional model of child and adolescent psychopathology: Tests using confirmatory factor analyses and multivariate behavior genetic analyses. Journal of Abnormal Psychology, 125, 1053. 27819467 10.1037/abn0000183 PMC6810679

[jcpp12849-bib-0047] Watamura, S.E. , Phillips, D.A. , Morrissey, T.W. , McCartney, K. , & Bub, K. (2011). Double jeopardy: Poorer social‐emotional outcomes for children in the NICHD SECCYD experiencing home and child‐care environments that confer risk. Child Development, 82, 48–65. 21291428 10.1111/j.1467-8624.2010.01540.x

[jcpp12849-bib-0048] Waters, E. , Stewart‐Brown, S. , & Fitzpatrick, R. (2003). Agreement between adolescent self‐report and parent reports of health and well‐being: Results of an epidemiological study. Child: Care, Health and Development, 29, 501–509. 10.1046/j.1365-2214.2003.00370.x 14616908

[jcpp12849-bib-0049] Wichstrøm, L. , Belsky, J. , & Steinsbekk, S. (2017). Homotypic and heterotypic continuity of symptoms of psychiatric disorders from age 4 to 10 years: A dynamic panel model. Journal of Child Psychology and Psychiatry, 58, 1239–1247. 28543077 10.1111/jcpp.12754

[jcpp12849-bib-0050] Willner, C.J. , Gatzke‐Kopp, L.M. , & Bray, B.C. (2016). The dynamics of internalizing and externalizing comorbidity across the early school years. Development and Psychopathology, 28(4pt1), 1033–1052. 27739391 10.1017/S0954579416000687 PMC5319409

