# Supplementary material for: Developmental stability of general and specific factors of psychopathology from early childhood to adolescence: dynamic mutualism or p‐differentiation?
Source: J Child Psychol Psychiatry. 2017 Dec 2;59(6):667–75. doi: 10.1111/jcpp.12849 (PMC6001631; doi:10.1111/jcpp.12849)
Supplement: Supplementary file 1 — Table S1. Means (SD), construct reliability (H), and standardized factor scores at age 2. Table S2. Means (SD), construct reliability (H), and standardized factor scores at age 3. Table S3. Means (SD), construct reliability (H), and standardized factor scores at age 5. Table S4. Means (SD), construct reliability (H), and standardized factor scores at age 6. Table S5. Means (SD), construct reliability (H), and standardized factor scores at age 8. Table S6. Means (SD), construct reliability (H), and standardized factor scores at age 9. Table S7. Means (SD), construct reliability (H), and standardized factor scores at age 10. Table S8. Means (SD), construct reliability (H), and standardized factor scores at age 11. Table S9. Means (SD), construct reliability (H), and standardized factor scores at age 14. [file JCPP-59-667-s001.pdf]

Table S1. Means (SD), construct reliability (*H*), and standardised factor scores at age 2

	p	INT	EXT	ATT
Mean (SD)		8.42 (5.74)	12.05 (6.18)	2.69 (1.62)
H	0.951	0.903	0.723	0.588
CBCL21	0.377	0.227		
CBCL46	0.368	0.431		
CBCL51	0.366	0.234		
CBCL79	0.260	0.362		
CBCL82	0.700	0.183		
CBCL83	0.547	0.309		
CBCL92	0.311	0.280		
CBCL97	0.575	-0.011		
CBCL99	0.333	0.596		
CBCL10	0.416	0.386		
CBCL33	0.324	0.304		
CBCL37	0.451	0.416		
CBCL43	0.473	0.453		
CBCL47	0.587	0.224		
CBCL68	0.280	0.379		
CBCL87	0.479	0.503		
CBCL90	0.385	0.640		
CBCL01	0.326	0.302		
CBCL07	0.131	0.265		
CBCL12	0.167	0.621		
CBCL19	0.310	0.130		
CBCL24	0.311	0.136		
CBCL39	0.178	0.532		
CBCL45	0.440	0.713		
CBCL52	0.107	0.592		
CBCL78	0.368	0.495		
CBCL86	0.214	0.434		
CBCL93	0.331	0.455		
CBCL02	0.384	0.210		
CBCL04	0.281	0.214		
CBCL23	0.513	0.125		
CBCL62	0.467	0.430		
CBCL67	0.522	0.372		
CBCL70	0.443	0.501		
CBCL71	0.388	0.561		
CBCL98	0.384	0.618		
CBCL08	0.563		0.359	
CBCL15	0.484		0.555	
CBCL16	0.552		0.476	
CBCL18	0.510		0.265	
CBCL20	0.470		0.636	

CBCL27	0.475	0.369	
CBCL29	0.632	0.075	
CBCL35	0.539	0.191	
CBCL40	0.480	0.376	
CBCL42	0.396	0.254	
CBCL44	0.739	0.005	
CBCL53	0.557	0.227	
CBCL58	0.509	0.374	
CBCL66	0.639	0.046	
CBCL69	0.534	0.254	
CBCL81	0.686	0.187	
CBCL85	0.559	0.261	
CBCL88	0.700	0.332	
CBCL96	0.665	0.086	
CBCL05	0.522		0.689
CBCL06	0.562		0.541
CBCL56	0.583		-0.028
CBCL59	0.567		0.302
CBCL95	0.331		-0.081

Table S2. Means (SD), construct reliability (*H*), and standardised factor scores at age 3

	p	INT	EXT	ATT
Mean (SD)		9.41(6.18)	11.74 (6.01)	2.61 (1.66)
H	0.951	0.872	0.750	0.628
CBCL21	0.479	0.243		
CBCL46	0.316	0.404		
CBCL51	0.301	0.357		
CBCL79	0.348	0.250		
CBCL82	0.733	0.184		
CBCL83	0.579	0.281		
CBCL92	0.339	0.345		
CBCL97	0.576	0.073		
CBCL99	0.336	0.394		
CBCL10	0.415	0.315		
CBCL33	0.297	0.315		
CBCL37	0.435	0.350		
CBCL43	0.487	0.446		
CBCL47	0.525	0.234		
CBCL68	0.316	0.312		
CBCL87	0.453	0.543		
CBCL90	0.500	0.446		
CBCL01	0.365	0.231		
CBCL07	0.177	0.224		
CBCL12	0.087	0.589		
CBCL19	0.279	0.169		
CBCL24	0.353	0.167		
CBCL39	0.234	0.387		
CBCL45	0.379	0.535		
CBCL52	0.118	0.607		
CBCL78	0.302	0.490		
CBCL86	0.240	0.387		
CBCL93	0.261	0.450		
CBCL02	0.389	0.058		
CBCL04	0.324	0.312		
CBCL23	0.520	0.182		
CBCL62	0.475	0.434		
CBCL67	0.436	0.453		
CBCL70	0.387	0.459		
CBCL71	0.403	0.507		
CBCL98	0.380	0.471		
CBCL08	0.567		0.284	
CBCL15	0.376		0.600	
CBCL16	0.579		0.375	
CBCL18	0.419		0.412	
CBCL20	0.523		0.502	

CBCL27	0.501	0.227	
CBCL29	0.673	0.145	
CBCL35	0.530	0.411	
CBCL40	0.383	0.565	
CBCL42	0.403	0.400	
CBCL44	0.681	0.173	
CBCL53	0.469	0.471	
CBCL58	0.548	0.291	
CBCL66	0.627	0.135	
CBCL69	0.514	0.234	
CBCL81	0.721	0.183	
CBCL85	0.619	0.264	
CBCL88	0.660	0.308	
CBCL96	0.688	0.073	
CBCL05	0.559		0.718
CBCL06	0.605		0.580
CBCL56	0.513		0.050
CBCL59	0.603		0.299
CBCL95	0.431		-0.140

Table S3. Means (SD), construct reliability (H), and standardised factor scores at age 5

	p	INT	EXT	ATT
Mean (SD)		5.10 (4.29)	7.05 (5.67)	1.811 (1.70)
H	0.960	0.808	0.817	0.649
CBCL14	0.513	0.110		
CBCL29	0.192	0.234		
CBCL30	0.395	0.146		
CBCL31	0.459	0.279		
CBCL32	0.243	0.438		
CBCL33	0.486	0.196		
CBCL35	0.482	0.346		
CBCL45	0.544	0.306		
CBCL50	0.525	0.458		
CBCL52	0.473	0.317		
CBCL71	0.342	0.448		
CBCL91	0.449	0.311		
CBCL112	0.369	0.537		
CBCL42	0.284	0.362		
CBCL65	0.512	0.245		
CBCL69	0.496	0.321		
CBCL75	0.159	0.483		
CBCL102	0.418	0.295		
CBCL103	0.607	0.394		
CBCL111	0.390	0.581		
CBCL47	0.325	0.157		
CBCL49	0.188	0.165		
CBCL54	0.532	0.251		
CBCL56A	0.427	0.304		
CBCL56B	0.375	0.336		
CBCL56C	0.446	0.442		
CBCL56D	0.168	0.140		
CBCL56E	0.283	0.171		
CBCL56F	0.383	0.413		
CBCL56G	0.334	0.295		
CBCL26	0.542		0.242	
CBCL39	0.469		0.058	
CBCL43	0.542		0.232	
CBCL63	0.457		-0.064	
CBCL67	0.317		-0.015	
CBCL73	0.244		-0.028	
CBCL81	0.513		0.263	
CBCL82	0.549		0.113	
CBCL90	0.461		0.317	
CBCL96	0.335		0.084	
CBCL106	0.498		0.173	

CBCL03	0.558	0.128	
CBCL16	0.610	0.254	
CBCL19	0.681	0.055	
CBCL20	0.510	0.732	
CBCL21	0.474	0.829	
CBCL22	0.629	0.354	
CBCL23	0.479	0.227	
CBCL37	0.591	0.246	
CBCL57	0.537	0.364	
CBCL68	0.657	0.172	
CBCL86	0.686	0.098	
CBCL87	0.727	0.070	
CBCL88	0.688	-0.192	
CBCL89	0.621	-0.122	
CBCL94	0.543	0.133	
CBCL95	0.632	0.193	
CBCL97	0.627	0.294	
CBCL104	0.639	0.067	
CBCL01	0.468		0.138
CBCL08	0.585		0.739
CBCL10	0.588		0.420
CBCL13	0.556		0.198
CBCL17	0.466		0.107
CBCL41	0.681		0.144
CBCL61	0.352		0.480
CBCL80	0.462		0.193

Table S4. Means (SD), construct reliability (H), and standardised factor scores at age 6

	p	INT	EXT	ATT
Mean (SD)		5.53 (4.44)	6.45 (5.49)	2.46 (2.28)
H	0.957	0.844	0.703	0.641
CBCL14	0.470	0.244		
CBCL29	0.216	0.349		
CBCL30	0.314	0.338		
CBCL31	0.374	0.297		
CBCL32	0.169	0.346		
CBCL33	0.513	0.150		
CBCL35	0.467	0.329		
CBCL45	0.515	0.264		
CBCL50	0.380	0.516		
CBCL52	0.420	0.429		
CBCL71	0.362	0.435		
CBCL91	0.433	0.247		
CBCL112	0.326	0.503		
CBCL42	0.279	0.309		
CBCL65	0.384	0.300		
CBCL69	0.431	0.257		
CBCL75	0.175	0.304		
CBCL102	0.349	0.253		
CBCL103	0.518	0.353		
CBCL111	0.380	0.451		
CBCL47	0.328	0.315		
CBCL49	0.188	0.239		
CBCL51	0.231	0.238		
CBCL54	0.447	0.281		
CBCL56A	0.157	0.447		
CBCL56B	0.278	0.429		
CBCL56C	0.241	0.660		
CBCL56D	0.096	0.226		
CBCL56E	0.280	0.283		
CBCL56F	0.235	0.579		
CBCL56G	0.279	0.506		
CBCL26	0.547		0.232	
CBCL39	0.401		0.246	
CBCL43	0.600		0.176	
CBCL63	0.430		-0.062	
CBCL67	0.546		0.018	
CBCL81	0.508		0.325	
CBCL82	0.315		0.417	
CBCL90	0.445		0.419	
CBCL106	0.362		0.421	
CBCL03	0.587		-0.147	

CBCL16	0.645	0.342	
CBCL19	0.639	-0.186	
CBCL20	0.675	0.446	
CBCL21	0.654	0.549	
CBCL22	0.741	0.110	
CBCL23	0.556	0.276	
CBCL37	0.595	0.328	
CBCL57	0.621	0.299	
CBCL68	0.687	-0.027	
CBCL86	0.664	-0.076	
CBCL87	0.607	-0.033	
CBCL88	0.645	-0.172	
CBCL89	0.476	0.129	
CBCL94	0.538	0.118	
CBCL95	0.654	-0.030	
CBCL97	0.530	0.407	
CBCL104	0.638	0.036	
CBCL01	0.387		0.237
CBCL08	0.563		0.577
CBCL10	0.601		0.343
CBCL13	0.454		0.542
CBCL17	0.348		0.402
CBCL41	0.689		0.106
CBCL61	0.484		0.374
CBCL80	0.442		0.484

Table S5. Means (SD), construct reliability (H), and standardised factor scores at age 8

	p	INT	EXT	ATT
Mean (SD)		5.63 (5.03)	5.99 (5.42)	2.48 (2.38)
H	0.963	0.863	0.786	0.649
CBCL14	0.581	0.145		
CBCL29	0.267	0.279		
CBCL30	0.328	0.314		
CBCL31	0.347	0.486		
CBCL32	0.152	0.521		
CBCL33	0.531	0.186		
CBCL35	0.556	0.429		
CBCL45	0.578	0.357		
CBCL50	0.485	0.517		
CBCL52	0.360	0.542		
CBCL71	0.395	0.512		
CBCL91	0.518	0.223		
CBCL112	0.329	0.593		
CBCL42	0.256	0.277		
CBCL65	0.462	0.244		
CBCL69	0.530	0.295		
CBCL75	0.170	0.423		
CBCL102	0.282	0.185		
CBCL103	0.581	0.465		
CBCL111	0.392	0.409		
CBCL47	0.311	0.285		
CBCL49	0.183	0.388		
CBCL51	0.231	0.410		
CBCL54	0.473	0.359		
CBCL56A	0.253	0.490		
CBCL56B	0.303	0.386		
CBCL56C	0.340	0.562		
CBCL56D	0.395	0.136		
CBCL56E	0.214	0.293		
CBCL56F	0.247	0.587		
CBCL56G	0.334	0.247		
CBCL26	0.567		0.263	
CBCL39	0.536		0.266	
CBCL43	0.617		0.287	
CBCL63	0.429		0.067	
CBCL67	0.309		-0.087	
CBCL81	0.367		0.365	
CBCL82	0.356		0.412	
CBCL90	0.534		0.405	
CBCL96	0.422		0.264	
CBCL03	0.576		0.185	

CBCL16	0.632	0.364	
CBCL19	0.622	0.020	
CBCL20	0.580	0.604	
CBCL21	0.574	0.697	
CBCL22	0.671	0.320	
CBCL23	0.520	0.391	
CBCL37	0.568	0.373	
CBCL57	0.534	0.387	
CBCL68	0.676	0.094	
CBCL86	0.771	-0.062	
CBCL87	0.798	-0.189	
CBCL88	0.731	-0.153	
CBCL89	0.626	0.058	
CBCL94	0.502	0.298	
CBCL95	0.704	0.148	
CBCL97	0.659	0.416	
CBCL104	0.609	0.206	
CBCL01	0.483		0.260
CBCL08	0.542		0.628
CBCL10	0.547		0.437
CBCL13	0.469		0.497
CBCL17	0.411		0.436
CBCL41	0.702		0.214
CBCL61	0.512		0.306
CBCL80	0.476		0.386

Table S6. Means (SD), construct reliability (H), and standardised factor scores at age 9

	p	INT	EXT	ATT
Mean (SD)		5.31 (4.91)	5.54 (5.16)	2.40 (2.39)
H	0.964	0.829	0.760	0.658
CBCL14	0.619	0.144		
CBCL29	0.318	0.348		
CBCL30	0.403	0.385		
CBCL31	0.413	0.407		
CBCL32	0.205	0.409		
CBCL33	0.611	0.125		
CBCL35	0.510	0.360		
CBCL45	0.626	0.284		
CBCL50	0.542	0.427		
CBCL52	0.356	0.500		
CBCL71	0.438	0.424		
CBCL91	0.568	0.122		
CBCL112	0.377	0.518		
CBCL42	0.434	0.236		
CBCL65	0.558	0.156		
CBCL69	0.574	0.054		
CBCL75	0.207	0.352		
CBCL102	0.413	0.270		
CBCL103	0.654	0.293		
CBCL111	0.494	0.377		
CBCL47	0.327	0.339		
CBCL49	0.294	0.307		
CBCL51	0.437	0.294		
CBCL54	0.411	0.286		
CBCL56A	0.375	0.414		
CBCL56B	0.327	0.369		
CBCL56C	0.377	0.606		
CBCL56D	0.298	0.165		
CBCL56E	0.281	0.143		
CBCL56F	0.286	0.553		
CBCL56G	0.357	0.464		
CBCL26	0.559		0.400	
CBCL39	0.451		0.329	
CBCL43	0.548		0.463	
CBCL63	0.405		0.167	
CBCL81	0.417		0.563	
CBCL82	0.335		0.252	
CBCL90	0.473		0.458	
CBCL96	0.532		0.181	
CBCL03	0.506		0.224	
CBCL16	0.622		0.338	

CBCL19	0.636	0.024	
CBCL20	0.537	0.405	
CBCL21	0.586	0.524	
CBCL22	0.622	0.343	
CBCL23	0.454	0.542	
CBCL37	0.561	0.291	
CBCL57	0.620	0.272	
CBCL68	0.684	0.102	
CBCL86	0.708	-0.034	
CBCL87	0.729	-0.072	
CBCL88	0.731	-0.124	
CBCL89	0.682	-0.008	
CBCL94	0.528	0.300	
CBCL95	0.696	0.176	
CBCL97	0.740	0.286	
CBCL104	0.595	0.035	
CBCL01	0.445		0.311
CBCL08	0.557		0.602
CBCL10	0.570		0.378
CBCL13	0.429		0.510
CBCL17	0.443		0.486
CBCL41	0.626		0.276
CBCL61	0.468		0.286
CBCL80	0.566		0.448

Table S7. Means (SD), construct reliability (H), and standardised factor scores at age 10

	p	INT	EXT	ATT
Mean (SD)		5.73 (5.06)	5.31 (5.35)	2.25 (2.43)
H	0.966	0.838	0.763	0.624
CBCL14	0.570	0.168		
CBCL29	0.333	0.300		
CBCL30	0.323	0.479		
CBCL31	0.413	0.392		
CBCL32	0.139	0.333		
CBCL33	0.642	0.141		
CBCL35	0.566	0.327		
CBCL45	0.612	0.233		
CBCL50	0.475	0.418		
CBCL52	0.489	0.452		
CBCL71	0.405	0.394		
CBCL91	0.535	0.206		
CBCL112	0.400	0.534		
CBCL42	0.402	0.198		
CBCL65	0.508	0.177		
CBCL69	0.505	0.212		
CBCL75	0.214	0.408		
CBCL102	0.458	0.235		
CBCL103	0.663	0.276		
CBCL111	0.481	0.236		
CBCL47	0.300	0.378		
CBCL49	0.316	0.301		
CBCL51	0.252	0.461		
CBCL54	0.449	0.299		
CBCL56A	0.304	0.498		
CBCL56B	0.258	0.498		
CBCL56C	0.357	0.602		
CBCL56D	0.202	0.169		
CBCL56E	0.216	0.225		
CBCL56F	0.286	0.591		
CBCL56G	0.286	0.350		
CBCL26	0.599		0.189	
CBCL39	0.551		0.310	
CBCL43	0.642		0.299	
CBCL63	0.476		0.096	
CBCL72	0.400		0.415	
CBCL81	0.500		0.543	
CBCL82	0.497		0.557	
CBCL90	0.347		0.384	
CBCL96	0.395		0.281	
CBCL03	0.688		0.028	

CBCL16	0.676	0.333	
CBCL19	0.639	-0.115	
CBCL20	0.611	0.495	
CBCL21	0.652	0.508	
CBCL22	0.720	0.209	
CBCL23	0.596	0.357	
CBCL37	0.562	0.268	
CBCL57	0.615	0.346	
CBCL68	0.627	-0.097	
CBCL86	0.731	-0.240	
CBCL87	0.728	-0.196	
CBCL88	0.686	-0.142	
CBCL89	0.654	-0.010	
CBCL94	0.544	0.200	
CBCL95	0.720	-0.006	
CBCL97	0.579	0.437	
CBCL104	0.619	0.073	
CBCL01	0.502		0.199
CBCL08	0.621		0.609
CBCL10	0.607		0.309
CBCL13	0.542		0.475
CBCL17	0.462		0.469
CBCL41	0.664		0.177
CBCL61	0.471		0.371
CBCL80	0.518		0.370

Table S8. Means (SD), construct reliability (H), and standardised factor scores at age 11

	p	INT	EXT	ATT
Mean (SD)		5.57 (5.17)	5.13 (5.23)	2.13 (2.30)
H	0.964	0.841	0.821	0.676
CBCL14	0.523	0.244		
CBCL29	0.289	0.331		
CBCL30	0.306	0.395		
CBCL31	0.487	0.348		
CBCL32	0.242	0.424		
CBCL33	0.640	0.162		
CBCL35	0.600	0.325		
CBCL45	0.576	0.256		
CBCL50	0.460	0.535		
CBCL52	0.423	0.500		
CBCL71	0.452	0.416		
CBCL91	0.483	0.268		
CBCL112	0.466	0.444		
CBCL42	0.371	0.347		
CBCL65	0.520	0.142		
CBCL69	0.544	0.113		
CBCL75	0.164	0.480		
CBCL102	0.519	0.232		
CBCL103	0.645	0.325		
CBCL111	0.521	0.362		
CBCL47	0.403	0.355		
CBCL49	0.176	0.260		
CBCL51	0.338	0.468		
CBCL54	0.417	0.328		
CBCL56A	0.372	0.389		
CBCL56B	0.353	0.381		
CBCL56C	0.386	0.634		
CBCL56D	0.326	0.061		
CBCL56E	0.241	0.227		
CBCL56F	0.328	0.523		
CBCL56G	0.239	0.425		
CBCL26	0.530		0.422	
CBCL39	0.474		0.427	
CBCL43	0.516		0.520	
CBCL63	0.448		0.204	
CBCL67	0.315		0.116	
CBCL72	0.350		0.204	
CBCL81	0.384		0.661	
CBCL82	0.366		0.555	
CBCL90	0.391		0.364	
CBCL96	0.421		0.146	

CBCL101	0.303	0.076	
CBCL03	0.650	0.211	
CBCL16	0.589	0.390	
CBCL19	0.650	-0.061	
CBCL20	0.616	0.443	
CBCL21	0.575	0.467	
CBCL22	0.609	0.418	
CBCL23	0.444	0.555	
CBCL37	0.579	0.278	
CBCL57	0.586	0.414	
CBCL68	0.543	0.261	
CBCL86	0.787	0.012	
CBCL87	0.771	-0.094	
CBCL88	0.793	-0.122	
CBCL89	0.624	0.047	
CBCL94	0.570	0.324	
CBCL95	0.675	0.271	
CBCL97	0.523	0.447	
CBCL104	0.586	0.193	
CBCL01	0.483		0.285
CBCL08	0.554		0.687
CBCL10	0.551		0.380
CBCL13	0.449		0.388
CBCL17	0.422		0.483
CBCL41	0.632		0.315
CBCL61	0.471		0.348
CBCL80	0.534		0.418

Table S9. Means (SD), construct reliability (H), and standardised factor scores at age 14

	p	INT	EXT	ATT
Mean (SD)		5.57(4.99)	4.90 (5.72)	2.09 (2.38)
H	0.970	0.845	0.778	0.619
CBCL14	0.535	0.130		
CBCL29	0.339	0.310		
CBCL30	0.383	0.286		
CBCL31	0.510	0.322		
CBCL32	0.175	0.198		
CBCL33	0.665	0.177		
CBCL35	0.571	0.314		
CBCL45	0.607	0.332		
CBCL50	0.501	0.410		
CBCL52	0.343	0.506		
CBCL71	0.398	0.381		
CBCL91	0.591	-0.020		
CBCL112	0.488	0.431		
CBCL42	0.372	0.226		
CBCL65	0.595	0.217		
CBCL69	0.579	0.145		
CBCL75	0.197	0.392		
CBCL102	0.506	0.336		
CBCL103	0.652	0.308		
CBCL111	0.384	0.407		
CBCL47	0.423	0.276		
CBCL49	0.283	0.303		
CBCL51	0.308	0.481		
CBCL54	0.397	0.384		
CBCL56A	0.323	0.432		
CBCL56B	0.317	0.491		
CBCL56C	0.373	0.633		
CBCL56D	0.228	0.091		
CBCL56E	0.234	0.262		
CBCL56F	0.183	0.676		
CBCL56G	0.161	0.494		
CBCL26	0.669		0.157	
CBCL39	0.565		0.388	
CBCL43	0.655		0.260	
CBCL63	0.440		0.163	
CBCL67	0.502		0.408	
CBCL72	0.402		0.315	
CBCL81	0.565		0.437	
CBCL82	0.576		0.533	
CBCL90	0.604		0.204	
CBCL96	0.526		0.159	

CBCL101	0.389	0.546	
CBCL105	0.410	0.521	
CBCL106	0.463	0.486	
CBCL03	0.647	-0.069	
CBCL16	0.644	0.191	
CBCL19	0.673	-0.005	
CBCL20	0.646	0.363	
CBCL21	0.666	0.390	
CBCL22	0.715	0.134	
CBCL23	0.601	0.388	
CBCL37	0.697	0.305	
CBCL57	0.617	0.291	
CBCL68	0.669	0.055	
CBCL86	0.777	-0.225	
CBCL87	0.745	-0.254	
CBCL88	0.727	-0.075	
CBCL89	0.638	0.188	
CBCL94	0.601	0.148	
CBCL95	0.720	0.115	
CBCL97	0.564	0.313	
CBCL104	0.644	0.073	
CBCL01	0.495		0.300
CBCL08	0.577		0.644
CBCL10	0.518		0.493
CBCL13	0.519		0.331
CBCL17	0.524		0.399
CBCL41	0.670		0.244
CBCL61	0.578		0.211
CBCL80	0.499		0.256
